# PIK3R3, a regulatory subunit of PI3K, modulates ovarian cancer stem cells and ovarian cancer development and progression by integrative analysis

**DOI:** 10.1186/s12885-022-09807-7

**Published:** 2022-06-27

**Authors:** Eun Jung Sohn

**Affiliations:** Pusan National University, Yangsan, 50612 Republic of Korea

**Keywords:** Ovarian cancer, Cancer stem cells, Phosphatidylinositol 3-kinase, Phosphoinositide-3-kinase regulatory subunit 3

## Abstract

**Background:**

Ovarian cancer is the most lethal gynecologic disease and is one of the most commonly diagnosed cancers among women worldwide. The phosphatidylinositol 3-kinase (PI3K) family plays an important regulatory role in various cancer signaling pathways, including those involved in ovarian cancer development; however, its exact function remains to be fully understood. We conducted this study to understand the role of P13K in the molecular mechanisms underlying ovarian cancer development.

**Methods:**

To determine the differential gene expression of phosphoinositide-3-kinase regulatory subunit 3 (*PIK3R3*), a regulatory subunit of PI3K, in normal, tumor, and metastatic ovary tissues, TNM plotter analysis was performed. The microarray dataset GSE53759 was downloaded from Gene Expression Omnibus. ROC plotter analysis was conducted to understand the potential of PIK3R3 as a predictive marker for effectiveness of therapy in ovarian cancer. muTarget was used to identify mutations that alter PIK3R3 expression in ovarian cancer. To determine the interacting partners for PIK3R3 in ovarian tissues, the interactome-atlas tool was used. The Kyoto encyclopedia of genes and genomes (KEGG*)* analysis was conducted to identify the pathways in which these interacting partners were primarily enriched.

**Results:**

PIK3R3 was overexpressed in ovarian and metastatic tumors. Elevated PIK3R3 levels were observed in ovarian cancer stem cells, wherein inhibiting PIK3R3 expression significantly reduced the size of ovarian cancer spheroids. Treatment of ovarian cancer stem cells with PF-04691502 (10 μM), an inhibitor of both PI3K and mTOR kinases, also reduced the size of spheroids and the level of *OCT4*. *PIK3R3* was highly expressed in ovarian cancer with several somatic mutations and was predicted better outcomes in patients undergoing Avastin® chemotherapy using bioinformatic tool. Protein interaction analysis showed that PIK3R3 interacts with 157 genes, including *GRB2, EGFR, ERBB3, PTK2, HCK, IGF1R, YES1*, and *PIK3CA*, in the ovary. KEGG enrichment analysis revealed that the interacting partners of PIK3R3 are involved in the ErbB signaling pathway, proteoglycans in cancer, FoxO, prolactin, chemokine, and insulin signaling pathways.

**Conclusions:**

PIK3R3 plays a pivotal role in ovarian cancer development and is therefore a potential candidate for developing novel therapeutic approaches.

**Supplementary Information:**

The online version contains supplementary material available at 10.1186/s12885-022-09807-7.

## Background

Ovarian cancer is the second most lethal gynecologic malignancy in the United States and the sixth most lethal malignancy in women in the western world [1]. Ovarian cancer is a heterogeneous disease, characterized by epithelial tumors originating from ovarian epithelial surface cells. Although the ovary consists of cells of different origins, > 90% of malignant tumors originate from ovarian epithelium [2]. Ovarian cancers that develop from epithelial cells represent distinct histological subtypes, namely, serous, mucinous, endometrioid, and clear cell carcinoma [3]. A high proportion 70% of the patients are diagnosed as having ovarian cancer only at the advanced stage of ovarian cancer given the vague disease symptoms [4]. Although tumor debulking surgery has improved with time, the survival rate of the disease remains low owing to the emergence of chemoresistance. Thus, it is important to identify molecular target for developing new and improved therapeutic strategies for epithelial ovarian cancer [5].

Cancer stem cells (CSCs) are capable of self-renewal and pluripotent differentiation, thereby facilitating tumorigenesis and cancer metastasis [6–8]. CSCs are the drivers of chemotherapy resistance, metastasis, and recurrence in several cancers, such as sarcomas and breast cancer [9, 10]. The pathways that modulate CSCs are upregulated in many aggressive tumors [11]. CSCs were first identified in the ascites of patients with ovarian cancer [12], and the inactivation of CSCs in ovarian cancer suppresses tumor progression and frequent relapses [13, 14].

Class IA phosphoinositide 3-kinases (PI3K) are heterodimeric enzymes with p110 catalytic and p85 regulatory subunits. p85α, p85β, and p55γ, isoforms of the p85 regulatory subunit, are encoded by *PIK3R1, PIK3R2*, and *PIK3R3*, respectively [15]. PIK3R3 is involved in inflammation, cell proliferation, and tumor growth [16–18]; it induces epithelial–mesenchymal transition (EMT) and promotes metastasis in colorectal cancer and prostate cancer cells [19, 20]. Additionally, PIK3R3 inhibits cell senescence and enhances cell proliferation via the p53/p21 signaling pathway [21]; it is highly expressed in ovarian cancer cells, and knocking down *PIK3R3* induces apoptosis in such cells [22, 23].

PIK3R3 is overexpressed in ovarian cancer cells; however, its exact function is not well understood. To understand the role of PIK3R3 in the molecular mechanisms underlying ovarian cancer development, we used bioinformatics approaches. We aimed to determine the potential of PIK3R3 as a predictive marker of ovarian cancer and to regulate PIK3R3 expression in somatically mutated ovarian cancer cells. We also aimed to deduce the role of PIK3R3 in the maintenance of stem cell properties in ovarian cancer cells. Our study shows that PIK3R3 is an important regulator of both ovarian cancer and ovarian CSCs.

## Methods

### Cell culture

The ovarian cancer cell lines A2780 and SKOV3 were obtained from the American Type Culture Collection (Rockville, MD, USA). The cells were cultured in RPMI 1640 medium (Thermo Fisher Scientific, Waltham, MA, USA) supplemented with 10% fetal bovine serum (FBS, Thermo Scientific) and 1% penicillin/streptomycin (Invitrogen Life Technologies, Carlsbad, CA, USA) and maintained at 37 °C in a humidified atmosphere under 5% CO_2_ conditions.

### Spheroid culture

Spheroid-forming CSCs were generated from A2780 and SKOV3 cells following a method as previously described [24]. Briefly, to isolate spheroid-forming cells, 80–90% *confluence* of A2780 and SKOV3 monolayers were detached using trypsin/EDTA solution (Thermo Scientific) and seeded in CSC culture medium containing Neurobasal™ medium (Thermo Scientific) with EGF (R&D Systems, Minneapolis, MN, USA), basic fibroblast growth factor (R&D Systems, Minneapolis, MN, USA), penicillin/streptomycin (Invitrogen Life Technologies, Carlsbad, CA, USA), Glutamax supplement (Thermo Scientific), B-27 supplement (Thermo Scientific), and HEPES (Sigma*-*Aldrich, St Louis, MO, USA) on ultra-low attachment 100-mm^2^ culture plates (Corning Inc., Corning, NY, USA).

### Gene expression omnibus data set analysis

We analyzed the Gene Expression Omnibus **(**GEO) dataset GSE53759 on ovarian cancer cell lines, including ovarian carcinoma IGROV-1 cell monolayer and spheroid-derived cells (*n* =3). To see differentially expressed genes between ovarian cancer and normal samples, GSE29450, GSE36668, GSE14001, and GSE69428 were analyzed. GSE124766 was used to see the level of *PIK3R3* in high-grade serous ovarian cancer (HGSOC) tumor organoids and tumor tissues. We then used GEO2R(http://www.ncbi.nlm.nih.gov/geo/geo2r/) to identify differentially expressed mRNAs in the abovementioned cell types of the dataset.

### *Real-Time *Quantitative* Reverse Transcription* PCR

Total RNA from monolayer cells (A2780 and SKOV3) and spheroid cells (A2780-SP and SKOV3-SP) was extracted using TRIzol® (Thermo Scientific) according to the manufacturer’s protocol. RNA (1 µg) was reverse-transcribed to cDNA using an RT First Strand kit (Cat. No. 330401, Qiagen, Valencia, CA, USA) and mixed with Power SYBR GREEN PCR Master Mix (Thermo Scientific) for r*eal*-*time* quantitative r*everse transcription* PCR (RT-qPCR) on an Applied Biosystems™ StepOne™ Real-Time thermal cycler (Thermo Scientific). The following primers were used: *PIK3R3* (forward primer 5′-ATG TAC AAT ACG GTG TGG AGT ATG-3′ and reverse primer 5′-GCT GGA TCC ATT TCA AT-3′); *GAPDH* (forward primer 5′-GAG AGA CCC TCA CTG CTG-3′ and reverse primer 5′-GAT GGT ACA TGA CAA GGT GC-3′); *OCT4* (forward primer 5′- TTTTGGTACCCCAGGCTATG ′ and reverse primer 5′- GCAGGCACCTCAGTTTGAAT-3′). All primers were purchased from Bionics (Seoul, Korea). For quantification purposes, *GAPDH* was used to normalize the mRNA level. The experiment was performed in triplicate.

### Western blotting

Proteins from monolayer cells (A2680 and SKOV3) and spheroid cells (A2780-SP, SKOV3-SP) were collected and extracted using RIPA buffer (Sigma-Aldrich). The Bio-Rad Protein Assay (Bio-Rad, Hercules, CA, USA) was used to determine protein concentration. The protein extract was separated using 10% sodium dodecyl sulfate–polyacrylamide gel electrophoresis and transferred onto a polyvinylidene difluoride membrane (Bio-Rad, Hercules, CA, USA). For blocking, the membrane was incubated with 8% (w/v) nonfat dry milk in PBS–Tween 20 (PBST; 0.05%, Sigma-Aldrich) at 4 °C overnight. After washing with PBST, the membranes were incubated with primary antibodies against PIK3R3 (1:1000, Abclonal, Woburn, MA, USA), OCT4 (1:1000, Abclonal), and GAPDH (1: 5000, Sigma-Aldrich) in PBST at 4 °C overnight. After washing with PBST, the membranes were incubated with HRP-conjugated secondary antibodies (Bio-Rad, Hercules, CA, USA) at room temperature for 1 h, and the protein bands were visualized via enhanced chemiluminescence (GE Healthcare Biosciences, Piscataway, NJ, USA).

### siRNA transfection

*PIK3R3* knockdown was performed with an siRNA using the following target sequences: sense (5′-GGA CUU GCU UUA UGG GAA A dTdT-3′) and antisense (3′-dTdT CCU GAA CGA AAU ACC CUU U-5′) (Bionic). The jetPRIME transfection reagent (Polyplus, New York, NY, USA) was used according to manufacturer's instructions. The number of tumor spheroids was determined after 3 and 5 days of transfection, and images depicting CSC morphology were acquired by using EVOS 7500 machine (Thermo Scientific). The experiments were repeated three times*.*

### Spheroid formation assay

Briefly, A2780-SP cells (2 × 10^3^) were seeded in 6-well ultra-low attachment plates (Corning) with or without 10 μM PF-04691502 (Sigma). After 7 days, the number of tumor spheroids was determined, and images were acquired by using EVOS 7500 machine (Thermo Scientific). The experiments were repeated three times*.*

### Gene correlation expression analysis

The starBase database (http:://starbase.sysu.edu.cn/, containing 32 The Cancer Genome Atlas [TCGA]-associated multidimensional datasets, including those for ovarian cancer) was used to study the correlation between the expression levels of *PIK3R3* and *SOX2* and other genes in TCGA ovarian cancer (OC) cohort. The results were analyzed statistically using the Pearson’s correlation coefficient.

### Comparison of protein expression levels

Human protein atlas (HPA; https://www.proteinatlas.org) was used to compare the expression levels of PIK3R3 in normal ovaries and ovarian carcinoma [25].

#### Comparison of gene expression levels

TNM plotter (https://www.tnmplot.com) enables real-time comparison of gene expression changes among normal, tumor, and metastatic tissues. This web tool was used to determine *PIK3R3* expression using TCGA datasets, and a direct comparison among normal, tumor, and metastatic tissues was conducted using the Mann–Whitney *U* test [26].

#### Receiver operating characteristic plotter for drug sensitivity

To determine the effect of PIK3R3 expression on potential anticancer drug treatment, we used available data from receiver operating characteristic (ROC) plotter (http://www.rocplot.org/), a transcriptome-level open-access database for biomarker validation and independent drug treatment response prediction. Samples with relapse-free survival at 6 months and pathological response to PIK3R3 (202743_at) were used, and the chemotherapy drug Avastin® was used for targeted therapy.

#### Correlation analysis between *PIK3R3* and somatic mutations

muTarget (https://www.mutarget.com/), a tool based on TCGA, provides correlations between mutations and expression of the gene of interest in cancer. The ‘Target’ analysis module was used to find mutations that alter the expression of target genes in ovarian cancer. The candidate gene *PIK3R3* was used as an input to determine the correlation between *PIK3R3* and somatic mutations in ovarian cancer.

#### protein–protein interaction data

To identify the interacting partners for PIK3R3 in ovarian tissues, we used the interactome-atlas tool (www.interactome-atlas.org) (Fig. 6A). Filter for tissue expression was selected with ovary.

#### Kyoto encyclopedia of genes and genomes anlysis

To determine the function of PIK3R3 interacting genes, we performed Kyoto encyclopedia of genes and genomes (KEGG) pathway enrichment analysis [27]. For analysis of KEGG pathway enrichment, we used the web-based DAVID v6.8 (https://david.ncifcrf.gov/tools.jsp).

#### Statistical analysis

All data are represented as the mean ± standard deviation of the mean (SD) and all the analyses were carried out at least three times. For the statistical comparisons, the Student's *t-*test was performed using GraphPad Prism 9 (GraphPad Software, La Jolla, CA, USA).

## Results

### *PIK3R3* expression in ovarian cancer

**Fig. 1:**
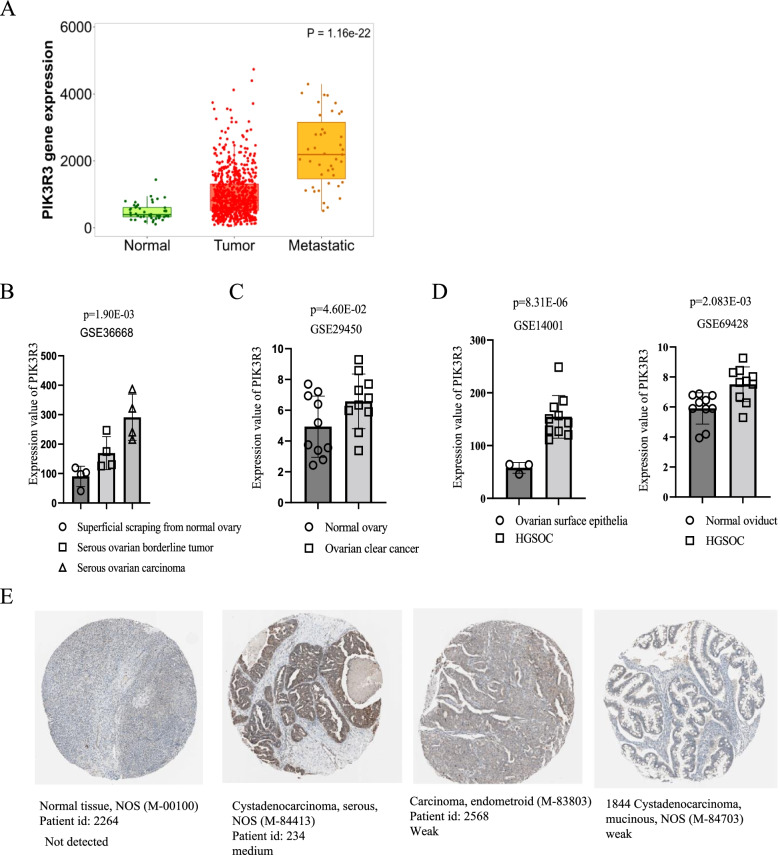
Differential transcript and protein expression of PIK3R3 in ovarian cancer. **A** *PIK3R3* transcript expression is significantly upregulated in tumors and metastatic tissues relative to normal tissues. *PIK3R3* expression obtained from the GSE36668 (**B**) and GSE29450 (**C**) dataset that contains information on serous ovarian carcinoma and ovarian clear cancer. **D** PIK3R3 expression level obtained from GSE14001 and GSE69428 datasets which contain information on HGSOC. **E** PIK3R3 protein expression in normal and ovarian cancer tissues. Courtesy of all immunohistochemical images: Human Protein Atlas, https://www.proteinatlas.org

Using TNM plotter, we determined the differential expression of *PIK3R3* in normal, tumor, and metastatic ovarian tissues. The expression of *PIK3R3* transcripts in both ovarian tumors and ovarian metastatic tumors was significantly higher than that in normal tissues (Fig. 1A).

In addition, using public datasets (GSE36668 and GSE29450), we observed that *PIK3R3* was highly expressed in serous ovarian cancer carcinoma (Fig. 1B) and ovarian clear cancer compared to normal ovary (Fig. 1C). *PIK3R3* was elevated in HGSOC compared to ovarian surface epithelia and normal oviduct using public datasets (GSE14001 and GSE69428) (Fig. 1D).

Next, to determine the protein levels of PIK3R3 in ovarian tumors, we searched the HPA for immunohistochemical images of normal, serous, and endometrioid cystadenocarcinoma tissues stained with antibodies against PIK3R3. As shown in Fig. 1E, ovarian carcinoma showed medium to low expression of PIK3R3, as documented by the HPA (https://www.proteinatlas.org/ENSG00000117461-PIK3R3/pathology/ovarian+cancer). Cystadenocarcinoma, serous, and endometrioid carcinoma tissues were stained “medium” or “weak” for PIK3R3, whereas no detectable intensity was observed for normal tissues (described as “not detected”), suggesting that PIK3R3 expression is specifically upregulated in ovarian carcinomas (Fig. 1E).

### ROC plotter analysis for chemotherapy and targeted therapy

**Fig. 2:**
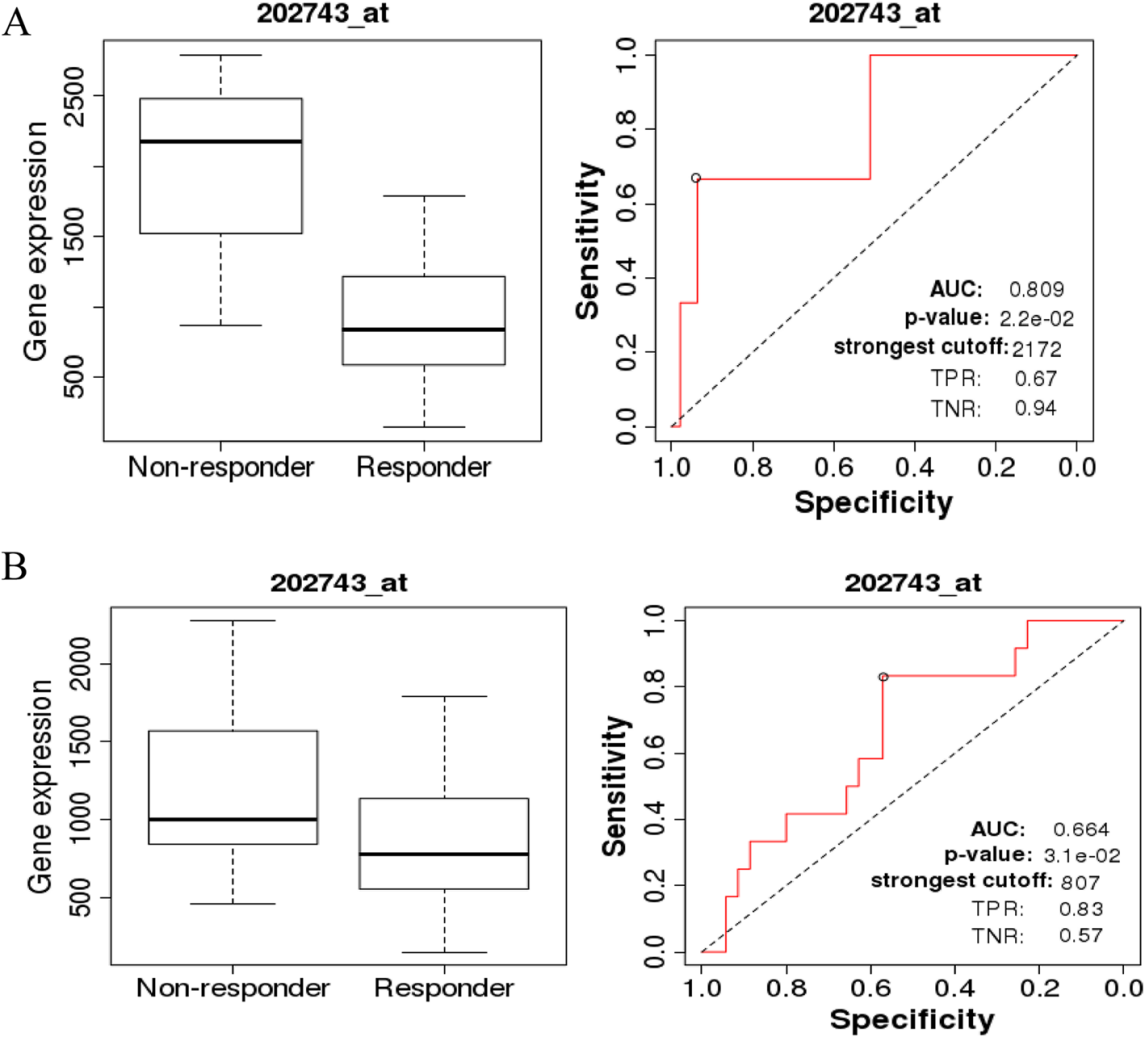
Receiver operating characteristic (ROC) curve of PIK3R3 in ovarian cancer patients. **A** ROC curves and boxplots of PIK3R3 in ovarian cancer patients undergoing Avastin® treatment for relapse-free survival time of 6 months. **B** ROC curves and boxplots of PIK3R3 in ovarian cancer patients undergoing Avastin® treatment for pathological response. Area under curve (AUC); TNR true negative rate (TNR); true positive rate (TPR) (http://www.rocplot.org/, accessed on October 2021)

To determine the potential of PIK3R3 as a predictive marker for therapy effectiveness in ovarian cancer, an ROC plotter analysis was performed to link gene expression to response to therapy. We compared PIK3R3 expression to Avastin® treatment in ovarian cancer specimens responders and non-responders. PIK3R3 is a relatively sensitive marker for predicting the effect of Avastin® treatment on patients with ovarian cancer based on relapse-free survival at 6 months (area under the curve [AUC] = 0.809, *P* = 2.2E-02) (Fig. 2A) and pathological response (AUC = 0.664, *P* = 3.1E-02) (Fig. 2B).

### Relationship between somatic mutations and *PIK3R3* expression

**Fig. 3:**
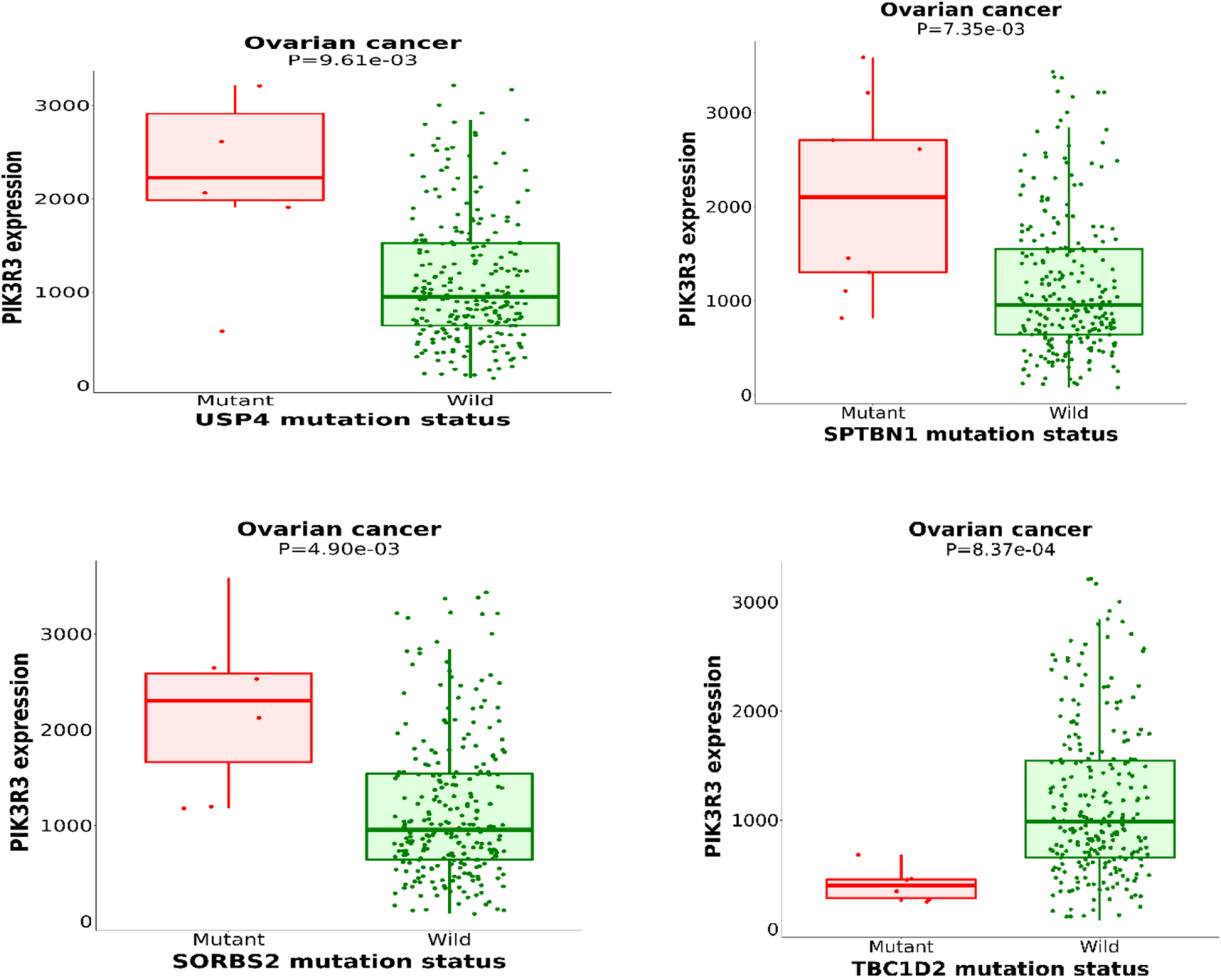
Association of *PIK3R3* expression with somatic mutations in ovarian cancer. Somatic mutations in the four genes *USP4, SPTBN1, SORBS*2, and *TBC1D2* are most strongly associated with alterations in *PIK3R3* expression in ovarian cancer. TARGET was used to determine somatic mutations in genes that significantly altered *PIK3R3* expression in ovarian cancer tissues. (https://www.mutarget.com/, accessed on October 2021)

Using muTarget, we identified somatic mutations influencing PIK3R3 expression. As shown in Fig. 3, Ubiqutin Specific Peptidase 4 (*USP4*)*,* pectrin Beta Chain, Non-Erythrocytic 1 (*SPTBN1*)*,* Sorbin And SH3 Domian Contanning 2 (*SORBS2*) and TBC1 Domain Family.

Member 2 (*TBC1D2*) strongly affected *PIK3R3* expression. *PIK3R3* expression was lower in *USP4*, *SPTBN1*, and *SORBS2*-mutant ovarian cancer patients than in ovarian cancer patients with wild type genes. However, *PIK3R3* expression was higher in tumor specimens having somatic mutations in *TBC1D2*.

**Fig. 4:**
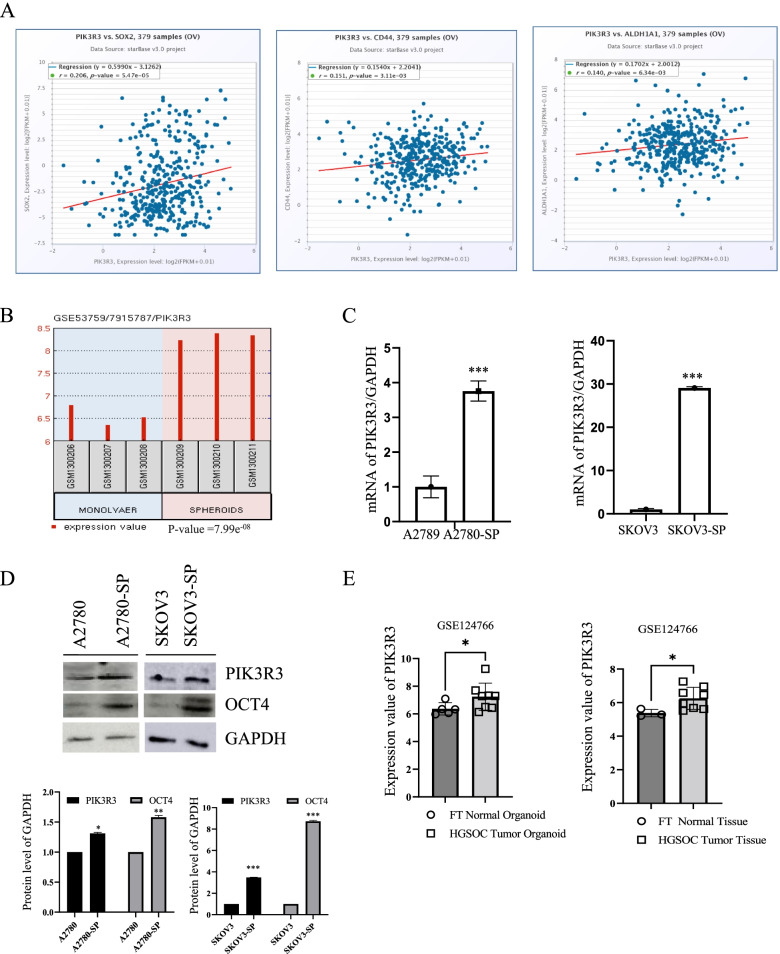
PIK3R3 overexpression in ovarian cancer stem cells. **A** Correlation analysis between PIK3R3 expression and expression of cancer stem cell-related proteins, such as SOX2, CD44, and ALDH1A1, in ovarian cancer stem cells. The starBase database was used to determine the correlation between *PIK3R3* and stem cell-related genes (*SOX2, CD44, ALDH1A*1). **B** PIK3R3 expression obtained from the GSE53759 dataset that contains information on monolayer and spheroid-derived cells from ovarian carcinoma IGROV-1 in triplicate. **C** RT-qPCR analysis of the mRNA levels of *PIK3R3* obtained from ovarian cancer (A2780 and SKOV3) and spheroid-derived (A2780-SP and SKOV3-SP) cells (*n* = 3). Data are represented as the mean ± SD. ***P < 0.001 vs control. **D** Western blot analysis of protein expression levels of PIK3R3 obtained from ovarian cancer (A2780 and SKOV3) and spheroid-derived (A2780-SP and SKOV3-SP) cells. Lower panels represented densitometric quantification of the western blots. Data are presented as the mean ± SD from three independent experiments. *P < 0.05; **P < 0.01; ***P < 0.001. **E** The PIK3R3 expression level obtained from the GSE124766 dataset that contains information on HGSOC tumor organoids and tumor tissues compared to FT normal organoids and FT Normal tissues. Data are presented as the mean ± SD from three independent experiments. *P < 0.05

To analyze the correlation between pluripotent genes and *PIK3R3*, starBase v2.0 was used. As shown in Fig. 4A, *PIK3R3* expression in ovarian cancer positively correlated with sex determining region Y-box 2 (*SOX2*), *CD44*, and aldehyde dehydrogenase 1 (*ALDH1A1*). These results suggest that *PIK3R3* is related to stem cells. To validate this, using the GEO database, we examined whether *PIK3R3* is expressed in ovarian CSCs. *PIK3R3* expression was significantly upregulated in ovarian CSCs compared with that in adherent cells (Fig. 4B). To confirm this finding, RT-qPCR was performed to compare *PIK3R3* mRNA from A2780 spheroid-derived (SP) cells and SKOV3-SP cells with that from A2780 and SKOV3 epithelial ovarian cancer cells (Fig. 4C). As shown in Fig. 4C, *PIK3R3* expression was significantly upregulated in A2780-SP and SKOV3-SP cells compared with that in adherent cells. Consistent with this, western blotting results showed that PIK3R3 level increased in both A2780-SP and SKOV3-SP cells (Fig. 4D). OCT4 used as a positive marker for cancer stem cells. We also checked the PIK3R3 level from HGSOC tumor organoids and HGSOC tumor tissues using public dataset (GSE124766). The level of the *PIK3R3* was elevated in the HGSOC tumor organoids and HGSOC tumor tissues compared to Fallopian tube (FT) normal organoids and FT normal tissues,respectively (Fig. 4E).

**Fig. 5:**
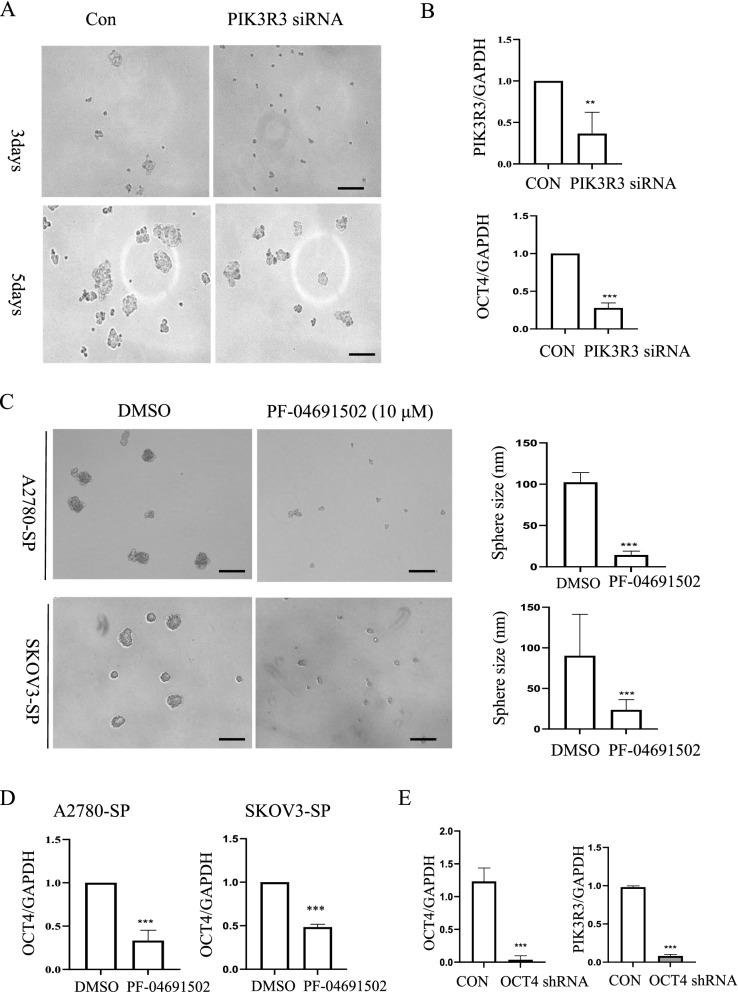
PIK3R3 is crucial for ovarian cancer spheroid-forming ability in vitro*.* **A** Reduction in the spheroid size of A2780-SP cells following siRNA-mediated *PIK3R3* knockdown. On 3 and 5 days after siRNA transfection on A2780-SP cells, the photo was taken under EVOS 7500. **B** The mRNA expression of OCT4 from PIK3R3 siRNA treated A2780-SP cells. The lysate after 3 days after transfection with *PIK3R3* siRNA was used for RT-qPCR to see the mRNA level of PIK3R3 and OCT4. Data are presented as the mean ± SD from three independent experiments. * **P < 0.01; ***P < 0.001*.* **C** Reduction in the spheroid size of A2780-SP and SKOV3-SP cells after PF-04691502 treatment. On 7 days from DMSO or PF-04691502 treated A2780-SP and SKOV3-SP, the morphology of sphere formation was taken under EVOS7500. Right panel showed the quantification of the sphere size (*n* ≥ 5). Data are presented as the mean ± SD from three independent experiments. **P < 0.01; ***P < 0.001*.* Scale bar = 200 nm. **D** The mRNA expression of OCT4 from A2780-SP and SKOV3-SP cells after PF-04691502 treatment. On 7 days from DMSO or PF-04691502 treated A2780-SP and SKOV3-SP, total lysate was used for RT-qPCR to see the mRNA level of OCT4. Data are presented as the mean ± SD from three independent experiments. ***P < 0.001. **E** The mRNA expression of PIK3R3 from OCT4 shRNA treated A2780 cells. The lysate after 3 days after transfection with OCT4 shRNA was used for RT-qPCR to see the mRNA level of PIK3R3 and OCT4. Data are presented as the mean ± SD from three independent experiments. **P < 0.01; ***P < 0.001

Additionally, *PIK3R3* knockdown using siRNA suppressed the spheroid-forming ability of A2780-SP cells (Fig. 5A). Also, silencing of *PIK3R3* knockdown using siRNA on A2780-SP repressed the mRNA level of OCT4 (Fig. 5B). Notably, treatment of A2780-SP cells with PF-04691502 (10 μM), an inhibitor of both PI3K and mTOR kinases, also reduced the size of spheroids (Fig. 5C) and the level of *OCT4* mRNA in A2780-SP and SKOV3-SP (Fig. 5D). In addition, knockdown of *OCT4* by using short hairpin RNA (shRNA) resulted in the reduction of *PIK3R3* mRNA expression in A2780 (Fig. 5E). Thus, our findings suggest that PIK3R3 is an important regulator of CSCs spheroid size.

### Interacting partners for PIK3R3 in ovarian tissues

**Fig. 6:**
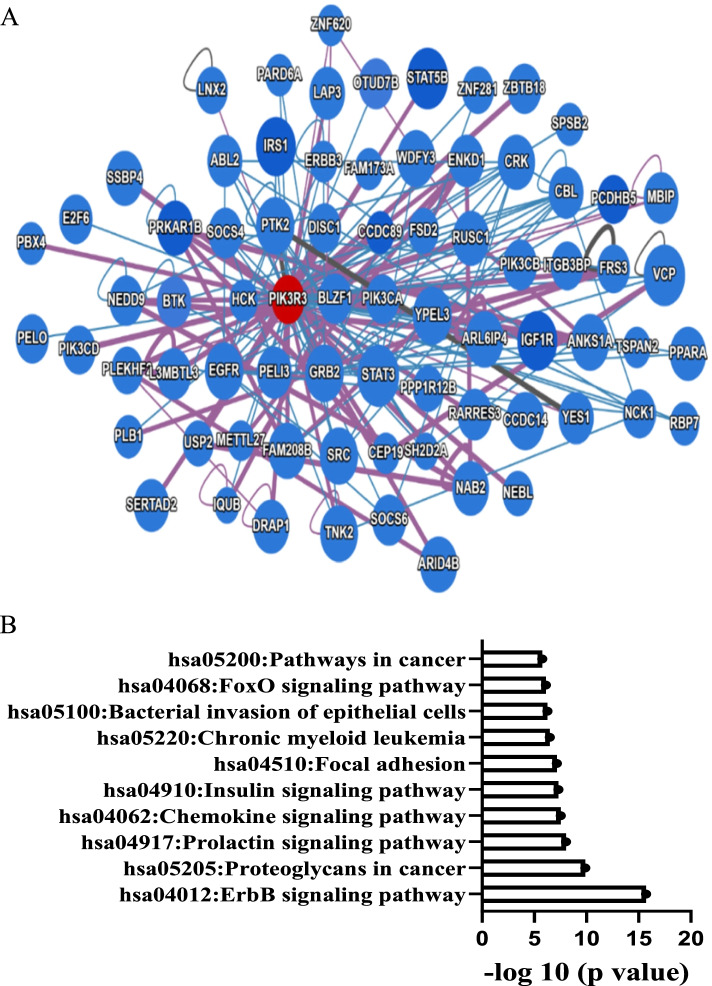
PIK3R3 interaction networks in ovarian tissues. **A** PIK3R3-interacting proteins in ovary determined using the interactome-atlas tool (www.interactome-atlas.org was accessed on 9 October 2021). **B** KEGG pathway enrichment analysis of PIK3R3 interacting protein partners. Kyoto encyclopedia of genes and genomes (KEGG) pathway enrichment analysis [27] was carreid out with PIK3R3 interacting genes

As shown in Fig. 6A, protein–protein interaction (PPI) analysis using the interactome-atlas tool showed that PIK3R3 interacted with 157 genes, including growth Factor Receptor Bound. Protein 2 (GRB2)*,* Epidermal Growth Factor Receptor (EGFR), Erb-B2 Receptor Tyrosine. Kinase 3 (ERBB3), Protein Tyrosine Kinase 2 (PTK2), HCK Proto-Oncogene, Src Family. Tyrosine Kinase (HCK), Insulin Like Growth Factor 1 Receptor(IGF1R), YES Proto. Oncogene 1, Src Family Tyrosine Kinase (YES1), and Phosphatidylinositol-4,5-Bisphosphate 3-Kinase Catalytic Subunit Alpha (PIK3CA). KEGG anaysis showed that proteins interacting with PIK3R3 were enriched in the ErbB signaling pathway, proteoglycans in cancer, prolactin signaling pathway, chemokine signaling pathway, insulin signaling pathway, focal adhesion, chronic myeloid leukemia, bacterial invasion of epithelial cells, FoxO signaling pathway, and pathways in cancer (Fig. 6B).

## Discussion

The PI3K/AKT signaling pathway is a key signaling pathway involved in cell survival, proliferation, and tumorigenesis. A recent study showed that the regulatory domain of PI3K, PIK3R3, plays an important role in maintaining sarcoma stem cells and promoting migration, invasion, and chemotherapy resistance [28]. PIK3R3 expression increases in ovarian cancer, whereas PIK3R3 deficiency results in apoptosis in ovarian cancer [23]. Consistent with this, our bioinformatics data analyses revealed that both mRNA and protein levels of PIK3R3 increased in ovarian cancer. In addition, similar results were observed for CSCs, where PIK3R3 levels were higher in ovarian CSCs and inhibition of PIK3R3 reduced their cellular size. Using muTAR analysis, PIK3R3 was highly expressed in several somatically mutated ovarian cancers and was predictive of ovarian cancer response to Avastin® chemotherapy. We also identified several interacting protein partners of PIK3R3 involved in diverse cellular pathways. Together, our findings support the position that PIK3R3 may be a crucial regulator of ovarian cancer development.

In this study, PIK3R3 was highly expressed in serous ovarian carcinoma and ovarian clear cancer compared to normal ovary using public datasets and was upregulated in ovarian cancer stem cells (A2780-SP and SKOV3-SP). We also showed that *PIK3R3* was elevated in the HGSOC tumor organoids and tissues using public datasets. Parida et al. reported that *PIK3R3* is upregulated in HGSOC compared to non-CSC tumor cells [29]. A previous study showed that the level of *PIK3R3* is significantly expressed in ovarian cancer cell lines including HGSOC cell lines (PEO1, OVCAR3, OVCAR4, OVCAR8, OVCAR10) than in human ovarian surface epithelial cells [23]. CSCs possess the stemness features such as of self-renewal and pluripotent differentiation [8, 30]. OCT4 is as an indispensable transcription factor that involves self-renewal and pluripotency in CSCs and is highly expressed in CSCs [31, 32]. The sphere-forming cells from ovarian cancer express stem-like properties and their CSC markers[33]. Previous study reported that PIK3R3 is upregulated in sarcoma CSCs and invovles in maintenances of CSC properties in sarcomas [28]. In this study, we showed that downregulation of PIK3R3 by siRNA or PF-04691502 treated ovarian CSC reduced the level of OCT4 and the spheroid-forming ability of ovarian CSC. Thus, our data imply that PIK3R3 is associated with stem-like properties of ovarian cancer cells.

Chemoresistance is a major problem in cancer therapeutics and is responsible for poor prognoses of ovarian cancer [34]. Thus, it is important to identify genes related to drug resistance in ovarian cancer to develop novel therapeutic strategies for this disease. Avastin® (bevacizumab), a humanized monoclonal antibody targeting vascular endothelial growth factor, has shown promising anticancer activity in an early phase II Gynecologic Oncology Group trial (protocol 170D) [35, 36]. In this study, we identified PIK3R3 as a potential biomarker for determining the effectiveness of Avastin® treatment and predicting progression-free survival in ovarian cancer patients. The AUC value for PIK3R3 was more than 0.8, which indicated that its anticancer drug response was satisfactory. Thus, our results suggest that PIK3R3 is a potential prognostic biomarker for Avastin®-mediated treatment of ovarian cancer.

Specific gene mutations contribute to the development and pathogenesis of ovarian cancer; gene mutations are related to the clinical phenotype of cancers, suggesting the critical role of the loci of gene mutations as prognostic and therapeutic targets [37–39]. *PIK3CA, TP53, BRCA1/2*, and *KRAS* are highly associated with epithelial ovarian cancer [39]. *PIK3CA* mutations are prevalent in ovarian clear cell carcinoma and endometrioid ovarian carcinoma [38]. In the present study, we observed that *PIK3R3* expression increased in ovarian tumors with somatically mutated *USP4, SPTBN1*, and *SORBS2* but decreased in those with somatically mutated *TBC1D2*, implying that *USP4, SPTBN1, TBC1D2*, and *SORBS2* mutations in ovarian cancer are closely related to *PIK3R3* expression.

Our PPI analysis showed that PIK3R3 interacts with EGFR and ERBB3 in the ovary. KEGG pathway analysis revealed that the interacting partners of PIK3R3 participated in the ErbB signaling pathway. The EGF/ErbB family of receptor tyrosine kinases, including EGFR, ERBB2, ERBB3, ERBB4, and 13 other polypeptide ligands, contain a conserved EGF domain involved in modulating cell growth of the ovarian surface epithelium [40]. Hence, overexpression of the EGFR/ErbB receptor family members facilitates the progression of epithelial ovarian cancer. Moreover, elevated levels of ERBB3 are associated with poor outcomes in ovarian cancer patients [41]. ERBB3 activates PI3K by promoting EMT in ovarian cancer cells [42]. Thus, our study suggests that PIK3R3 affects ovarian cancer via the ErbB signaling pathway. It needs to further study the relationship between PIK3R3 and ErbB signaling in ovary cancer.

## Conclusions

PIK3R3 expression is upregulated in ovarian cancer as well as ovarian CSCs. Inhibition of PIK3R3 reduced the spheroid size of ovarian CSCs. ROC plotter analysis of drug responses showed that PIK3R3 may be a potential predictive biomarker for Avastin®-mediated ovarian cancer treatment. PIK3R3 expression was altered in cases of ovarian cancers with somatic mutations in *USP4, SPTBN1, SORBS2*, and *TBC1D2*. Collectively, our results suggest that PIK3R3 is a useful prognostic marker for ovarian cancer and can therefore be exploited for developing therapeutic strategies for this cancer type.

## Supplementary Information

**Additional file 1.**

## Data Availability

The datasets GSE29450, GSE36668, GSE14001, GSE69428, and GSE124766 for this study can be found in the GEO database (https://www.ncbi.nlm.nih.gov/geo/) and are available in the NCBI-GEO repository. https://www.ncbi.nlm.nih.gov/geo/query/acc.cgi?acc=GSE29450 https://www.ncbi.nlm.nih.gov/geo/query/acc.cgi?acc=GSE36668 https://www.ncbi.nlm.nih.gov/geo/query/acc.cgi?acc=GSE14001 https://www.ncbi.nlm.nih.gov/geo/query/acc.cgi?acc=GSE69428 https://www.ncbi.nlm.nih.gov/geo/query/acc.cgi?acc=GSE124766

## Abbreviations

AUC: Area under the curve
CSC: Cancer stem cell
FBS: Fetal bovine serum
GEO: Gene Expression Omnibus
HPA: Human Protein Atlas
KEGG: Kyoto encyclopedia of genes and genomes
ROC: Receiver operating characteristic
