# Supplementary material for: PIK3R3, a regulatory subunit of PI3K, modulates ovarian cancer stem cells and ovarian cancer development and progression by integrative analysis
Source: BMC Cancer. 2022 Jun 27;22:708. doi: 10.1186/s12885-022-09807-7 (PMC9238166; doi:10.1186/s12885-022-09807-7)
Supplement: Supplementary file 1 — Additional file 1. [file 12885_2022_9807_MOESM1_ESM.pptx]

## Slide 1
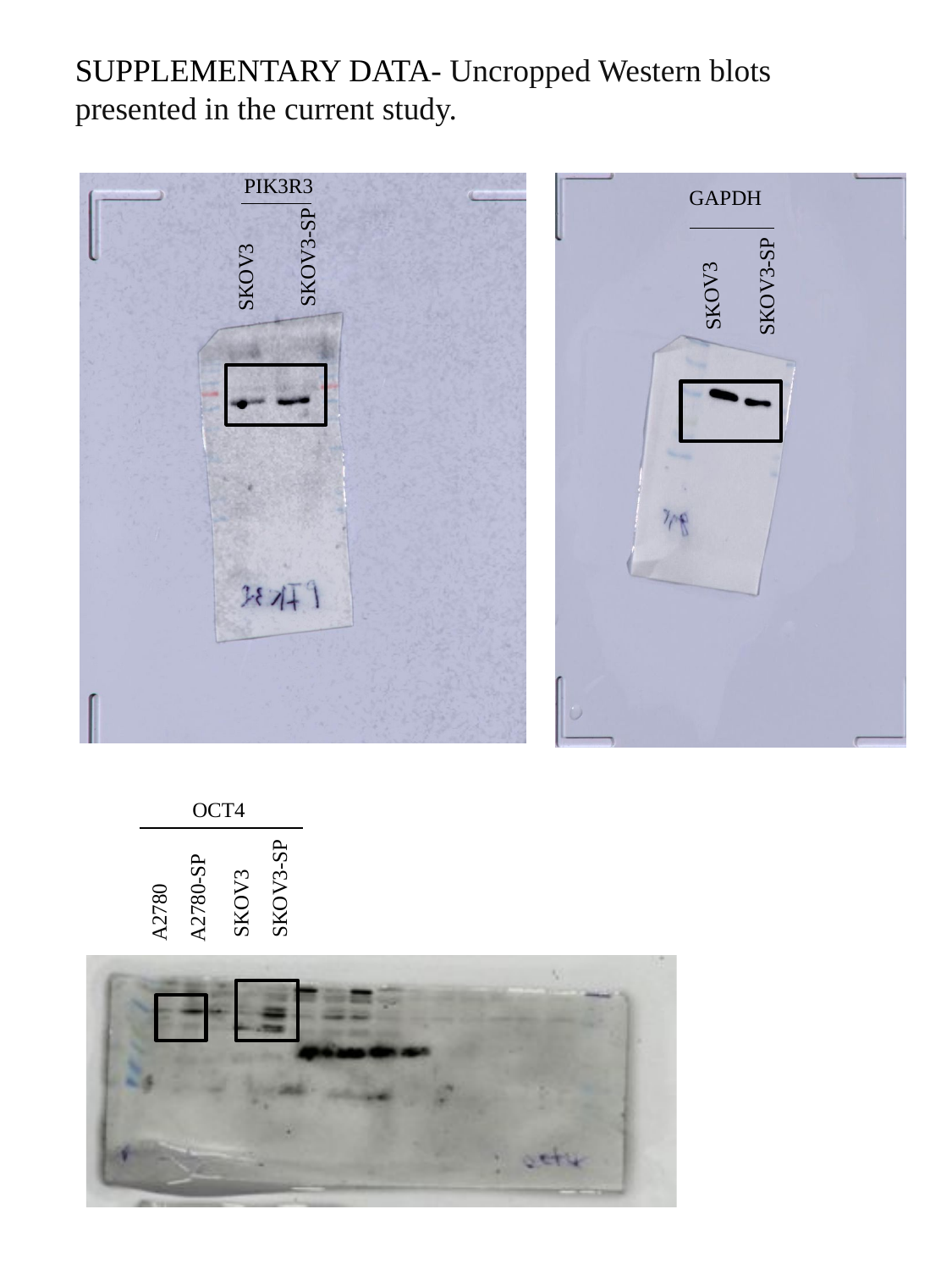

SUPPLEMENTARY DATA- Uncropped Western blots presented in the current study.
PIK3R3
GAPDH
SKOV3-SP
SKOV3
SKOV3-SP
SKOV3
OCT4
SKOV3-SP
A2780-SP
SKOV3
A2780

## Slide 2
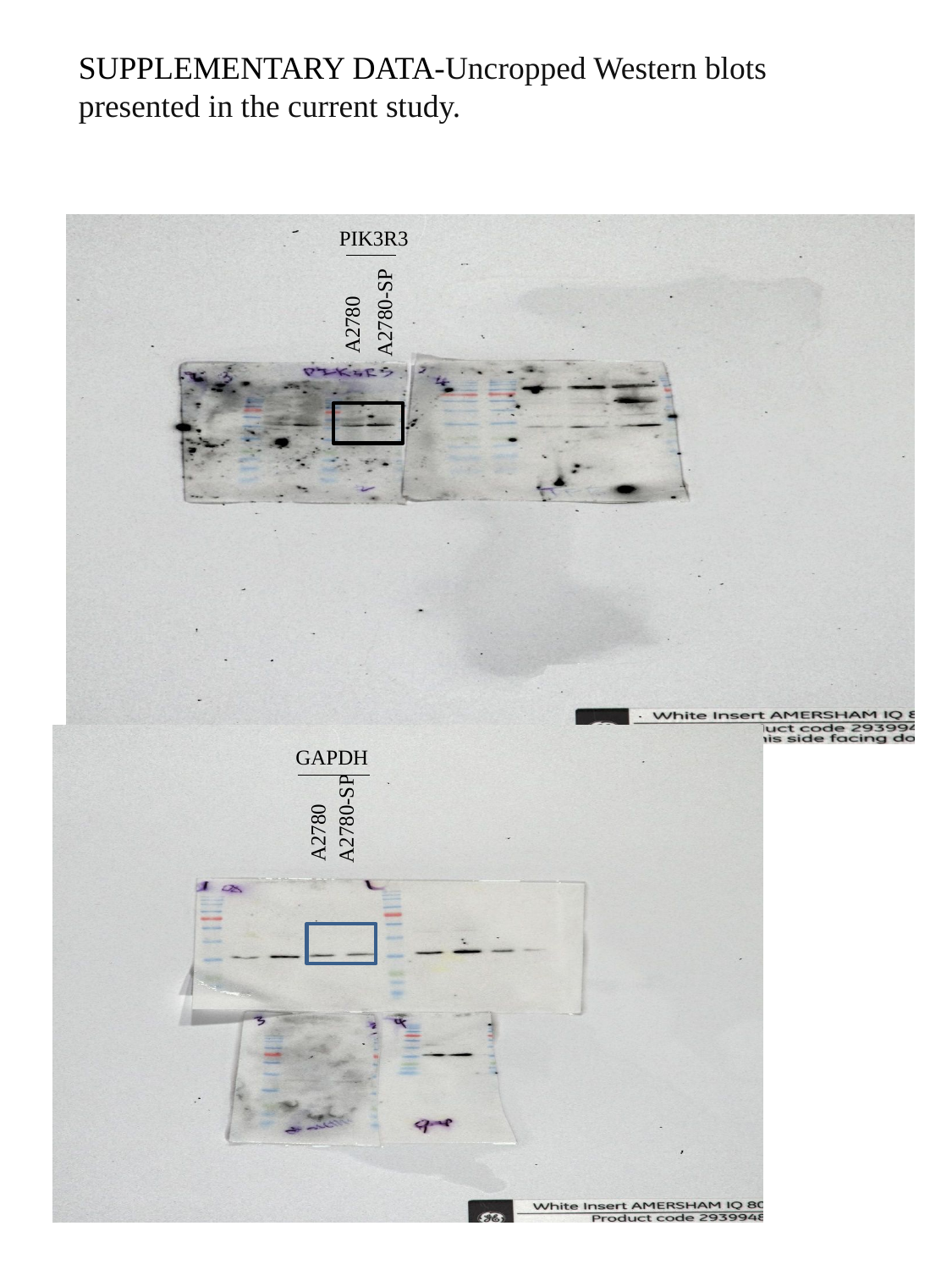

SUPPLEMENTARY DATA-Uncropped Western blots presented in the current study.
PIK3R3
A2780-SP
A2780
GAPDH
A2780-SP
A2780
